# Discordance in HER2 status between primary gastric adenocarcinoma tumors and cells from the corresponding malignant effusions

**DOI:** 10.1186/s12885-019-6035-0

**Published:** 2019-09-03

**Authors:** Hongsik Kim, Seung-Myoung Son, Chang Gok Woo, Ok-Jun Lee, Dae Hoon Kim, Hyo Yung Yun, Jieun Yun, Hee Kyung Kim, Yaewon Yang, Hye Sook Han

**Affiliations:** 1 Department of Internal Medicine, Chungbuk National University Hospital, Chungdae-ro 1, Seowon-gu, Cheongju, 28644 South Korea; 2 Department of Pathology, Chungbuk National University Hospital, Cheongju, South Korea; 3 Department of Pathology, Chungbuk National University College of Medicine, Cheongju, South Korea; 4 Department of Surgery, Chungbuk National University Hospital, Cheongju, South Korea; 5 Department of Surgery, Chungbuk National University College of Medicine, Cheongju, South Korea; 6 Department of Pharmaceutical Engineering, College of Science & Engineering, Cheongju University, Cheongju, South Korea; 7 Department of Internal Medicine, Chungbuk National University College of Medicine, Cheongju, South Korea

**Keywords:** Gastric adenocarcinoma, HER2, Malignant effusion

## Abstract

**Background:**

Metastasis of gastric cancer commonly manifests as a malignant effusion, which presents an alternative cell source for human epidermal growth factor receptor 2 (HER2) status identification. This study aimed to compare HER2 status in primary gastric adenocarcinoma tumors and corresponding cell blocks prepared from malignant effusions (CB-MEs).

**Methods:**

HER2 status was retrospectively evaluated by immunohistochemistry (IHC) in primary gastric adenocarcinomas and paired pathologically confirmed CB-MEs of 45 patients. Silver in situ hybridization (SISH) was also performed in cases with IHC 2+ for primary gastric adenocarcinomas and above IHC 1+ for CB-MEs.

**Results:**

HER2 positivity was observed in 4.4% (2/45) of primary gastric adenocarcinomas and 6.7% (3/45) of CB-MEs. The HER2 concordance rate between primary gastric adenocarcinomas and CB-MEs was 88.9% (40/45) (κ = − 0.056). All five patients with HER2 positivity in the primary tumor or a CB-ME had a negative result in the corresponding paired sample. Of the 15 patients with two or more serially sampled CB-MEs, HER2 expression determined by IHC differed between each CB-ME in six (40%) patients, and all three patients with HER2 positivity in CB-MEs exhibited HER2 positivity in one of the serially sampled CB-MEs.

**Conclusions:**

The HER2 positivity rate was very low in gastric cancer patients with malignant effusions. Our results suggest that HER2 positivity was discordant between the primary gastric adenocarcinoma and corresponding CB-MEs and among serially sampled CB-MEs. The possibility of detecting HER2 positivity can be improved if the primary gastric adenocarcinoma tumor as well as all the available CB-MEs from each patient are analyzed.

## Background

For unresectable locally advanced or metastatic gastric or gastroesophageal junction (GEJ) adenocarcinomas, the prognosis is dismal [1]. The 5 year survival rate is approximately 5–20%, and overall survival is ~ 10 months for those who receive conventional chemotherapy [1, 2]. Recently, molecular-targeted therapy has been proven to improve the survival of advanced gastric or GEJ cancer patients. As in breast cancer, the transmembrane tyrosine kinase receptor human epidermal growth factor receptor 2 (HER2) is now a well-established therapeutic target in gastric cancer [3]. A recent, randomized phase III trial demonstrated that the anti-HER2 monoclonal antibody trastuzumab, in combination with standard chemotherapy, significantly prolonged survival for patients with unresectable or metastatic HER2-positive gastric or GEJ cancer compared with chemotherapy alone [4]. Based on these results, trastuzumab and chemotherapy have become the new standard of treatment for patients with unresectable or metastatic HER2-positive gastric or GEJ cancer, and HER2 status has become an established predictive biomarker for selecting patients eligible for HER2-targeted therapy.

HER2 protein overexpression and/or gene amplification (HER2 positivity) is found in approximately 13–22% of gastric or GEJ cancers [4–8]. However, unlike breast cancers, gastric cancers frequently show incomplete membrane staining by immunohistochemistry (IHC), and HER2 heterogeneity has been described to range from 4.8% to up to 50% of cases [7–9]. The heterogeneity in HER2 expression in gastric cancers can potentially give rise to discordant results between different samples obtained from the same patient, leading to false-negative interpretation and potential undertreatment. It is also important to note that HER2 status is usually assessed in primary gastric tumors and that is the result used to guide therapy for recurrent or metastatic disease; however, discordance in HER2 positivity between primary and metastatic tumors developing either synchronously or metachronously could be misleading. Thus, the intra- or inter-tumoral heterogeneity of HER2 positivity in gastric cancers can present a major challenge when seeking to identify those patients who could benefit from HER2-targeted therapy.

Peritoneal carcinomatosis is the relatively common manifestation of gastric cancer metastasis, and up to 40% of patients have peritoneal spread causing ascites [10]. Patients with peritoneal carcinomatosis and ascites have a very poor prognosis, and peritoneal carcinomatosis is responsible for ~ 60% of gastric cancer-associated mortalities [11]. Furthermore, the presence of malignant ascites can be particularly painful and life threatening, severely affecting quality of life [12]. Sampling of malignant effusions such as ascitic or pleural fluid is a relatively simple, minimally invasive, and repeatable procedure, and these cytologic materials can be concentrated and stored as cell blocks, and stained as for a tissue sample. Cell blocks prepared from malignant effusions (CB-MEs) are, therefore, a feasible source for HER2 assessment in gastric cancer patients.

The purpose of this study was to investigate HER2 status in CB-MEs from gastric cancer patients and to assess the HER2 status concordance rate between primary gastric adenocarcinomas and corresponding CB-MEs.

## Methods

### Patients and sample collection

Cases of gastric adenocarcinoma with archived cell blocks of ascitic or pleural fluid seen at Chungbuk National University Hospital between June 2009 and May 2017 were reviewed from the pathologic database. The CB-MEs were prepared by centrifuging the serous fluid specimens for 10 min at 1800 rpm. After discarding the supernatant, the pellet was resuspended in 95% ethanol and centrifuged again. The pellet was then cut to a suitable size, and transferred to a tissue-embedding cassette. As for tissue processing, the CB-ME was obtained by subjecting the embedded cell pellet to three changes of alcohol, two changes of xylene, and two changes of paraffin wax. Sections (4 μm thick) were cut using a microtome (Leica Biosystems RM 2245, Nussloch, Germany) and stained with hematoxylin and eosin.

All samples contained adenocarcinoma cells. Tumor cellularity of CB-MEs was graded, according to the percentage of tumor cells, as low (≤10% tumor cells) or high (> 10%). Paired formalin-fixed, paraffin-embedded surgically resected or endoscopic biopsy specimens of primary gastric adenocarcinoma were retrieved from the archive for comparison. Only patients with pathologically confirmed gastric adenocarcinoma who showed no evidence of another malignant tumor and who had paired samples of CB-MEs and primary gastric adenocarcinomas stored were selected for this study. The HER2 status of the primary tumor was determined in surgically resected specimens where available, or otherwise in biopsy specimens.

This study was a retrospective analysis of leftover specimens that were discarded from general medical care or analysis. The requirement for participants’ consent was waived according to the Standard Operation Procedure of the Institutional Review Board of Chungbuk National University Hospital. The study, including the retrospective use of archival CB-MEs or tissues for HER2 assessment and consent exemption for study participants, was reviewed and approved by the Institutional Review Board of Chungbuk National University Hospital.

### HER2 assessment

HER2 status was determined in the pathological samples by IHC and silver in situ hybridization (SISH). IHC was performed with anti-HER2/neu rabbit monoclonal primary antibody (clone 4B5; Ventana Medical Systems, Inc., Tucson, AZ, USA) using a BenchMark XT autostainer (Ventana Medical Systems, Inc.), according to the manufacturer’s instructions. HER2 was scored according to the established modified scoring criteria for gastric cancer [13, 14]. Briefly, for biopsy specimens, 0 was defined as no membranous reactivity in any tumor cell; 1+ as the presence of a tumor cell cluster (≥5 cells) with faint membranous reactivity; 2+ as the presence of a tumor cell cluster (≥5 cells) with weak to moderate complete, basolateral, or lateral membranous reactivity, and 3+ as the presence of a tumor cell cluster (≥5 cells) with strong complete, basolateral, or lateral membranous reactivity. For resected specimens, 0 was defined as no staining or < 10% tumor cell positive staining; 1+ as faintly staining the membrane of ≥10% of tumor cells and part of their membrane; 2+ as weak to moderate complete, basolateral, or lateral membranous reactivity in ≥10% of tumor cells, and 3+ as strong complete, basolateral, or lateral membranous reactivity in ≥10% of tumor cells. For CB-ME specimens, the IHC findings were scored according to the criteria described for biopsy specimens. SISH was performed using Inform HER2 DNA and chromosome 17 (CEP17) probes (Ventana Medical Systems, Inc.) and detected using two-color chromogenic ISH on formalin-fixed, paraffin-embedded specimens. The two probes were sequentially hybridized on one slide. The specimen was then counterstained with Harris hematoxylin. The entire slide was scanned for areas of *HER2* amplification, using the HER2 IHC slide as a guide, and the *HER2* (appearing as a black dot) and CEP17 (appearing as a red dot) signals were counted in 20 tumor nuclei by light microscopy. The total copy-number count for *HER2* was divided by the total copy number for CEP17, and a score of 2 or more was considered amplified [8]. In addition to the *HER2*:CEP17 ratio, if > 6 copies of the *HER2* gene were detected with the HER2 single probe then the sample was also considered to have amplified *HER2* [8]. SISH was only performed on primary gastric adenocarcinoma tissue scored as IHC 2+, but was performed on all of the HER2 stained (IHC 1+, 2+, and 3+) CB-MEs.

HER2 positivity in the primary gastric adenocarcinomas was defined as IHC 3+, or IHC 2+ and *HER2* amplification by SISH. HER2 positivity in the CB-ME samples was defined as IHC3+ or *HER2* amplification by SISH because there was a possibility of false-negative interpretation due to low cellularity in the cytologic samples. The H&E sections and HER2 status of all of the cases were reviewed by two independent experienced pathologists without knowledge of the corresponding clinicopathological data. In cases of disagreement, HER2 status was eventually determined by consensus after re-examination.

### Statistical analysis

The rate of concordance in HER2 status between the primary gastric adenocarcinomas and paired CB-MEs was calculated as the ratio of concordant cases to total cases. The level of concordance in HER2 status was also evaluated using Cohen’s kappa coefficient (κ-value) statistic [15]. The level of concordance was classified according to the following outcomes: poor (κ < 0.00), slight (κ = 0.00–0.20), fair (κ = 0.21–0.40), moderate (κ = 0.41–0.60), substantial (κ = 0.61–0.80), or almost perfect (κ = 0.81–1.00). The statistical analyses were performed using IBM SPSS Statistics, software version 21.0 (IBM Corp., Armonk, NY, USA).

## Results

### Clinicopathological characteristics

Of the 130 cases of primary gastric adenocarcinoma with CB-MEs identified from the database review, 45 also had paired surgically resected or endoscopic biopsy specimens of primary gastric adenocarcinoma available in the archive. The clinicopathological characteristics of the 45 patients in this study are summarized in Table 1. Primary gastric adenocarcinoma tissue samples consisted of 38 endoscopic biopsy specimens (84.4%) and seven surgically resected specimens (15.6%). Lauren diffuse type was present in 23 patients (51.1%), and poorly differentiated tubular adenocarcinoma or poorly cohesive carcinoma was present in 24 (53.3%). From the 45 patients, there were 71 CB-MEs, 64 (90.1%) derived from ascitic fluid and seven (9.9%) from pleural fluid. Tumor cellularity in the CB-MEs was estimated to be high (> 10%) in 39 cases (54.9%) and low (≤10%) in 32 (45.1%). Forty-eight CB-MEs (67.6%) were obtained during or after palliative chemotherapy for unresectable or metastatic gastric adenocarcinoma, and the remainder were obtained at the time of diagnosis of metastatic cancer.

**Table 1:** Patient characteristics

	No. of patients (*n* = 45)		
	No.		%
Age at diagnosis, years			
Median	60 (range: 32 to 81)		
Sex			
Male	30		66.7
Female	15		33.3
Primary tumor location			
Gastroesophageal junction/fundus	4		8.9
Body	8		17.8
Antrum	22		48.9
Diffuse	11		24.4
Primary tumor tissue for HER2 assessment			
Endoscopic biopsy	38		84.4
Surgical resection	7		15.6
Lauren classification			
Intestinal	21		46.7
Diffuse	23		51.1
Mixed	1		2.2
WHO classification			
Tubular adenocarcinoma			
Well differentiated	1		2.2
Moderately differentiated	18		40.0
Poorly differentiated	13		28.9
Mucinous carcinoma	1		2.2
Poorly cohesive carcinoma	11		24.5
Mixed adenocarcinoma	1		2.2
Cell block of malignant effusion (*n* = 71)			
Sample type			
Ascitic fluid	64		90.1
Pleural fluid	7		9.9
Cellularity			
High (> 10% tumor cells)	40		56.3
Low (≤10% tumor cells)	31		43.7
Time of effusion sample collection			
At the diagnosis of metastatic disease before chemotherapy	23		32.4
During or after palliative chemotherapy	48		67.6

HER2, human epidermal growth factor receptor 2; WHO, World Health Organization

### HER2 status in primary gastric adenocarcinomas and CB-MEs

The HER2 status of primary gastric adenocarcinoma tumor tissue samples and CB-MEs is shown in Table 2. In patients with serially sampled CB-MEs, the CB-ME sample with the highest IHC score was selected and compared with the primary tumor. HER2 expression was detected by IHC in 35.6% (16/45) of primary gastric adenocarcinomas and 22.2% (10/45) of CB-MEs. The HER2 status of the primary gastric adenocarcinomas was scored as 0 in 29 specimens (64.4%), 1+ in seven (15.6%), 2+ in seven (15.6%), and 3+ in two (4.4%), while CB-MEs were scored as 0 in 35 specimens (77.8%), 1+ in six (13.3%), 2+ in one (2.2%), and 3+ in three (6.7%). The *HER2* SISH assay was carried out on the seven primary gastric adenocarcinomas that scored IHC 2+ and on the 10 CB-MEs that scored IHC 1+ to 3+, but amplified *HER2* was found only in the three CB-MEs that scored IHC 3 + .

**Table 2:** Comparison of HER2 status between primary gastric adenocarcinomas and cell blocks of malignant effusions

HER2 IHC score in the primary gastric adenocarcinoma tissue		HER2 IHC score in the malignant effusion cell blocks				Total
		Negative			Positive	
		0	1	2	3	
Negative	0	24	4	0	1^*^	29 (64.4%)
	1	5	1		1^*^	7 (15.6%)
	2	5		1	1^*^	7 (15.6%)
Positive	3	1	1			2 (4.4%)
Total		35 (77.8%)	6 (13.3%)	1 (2.2%)	3 (6.7%)	45 (100.0%)

*HER2*, Human epidermal growth factor receptor 2; *IHC*, Immunohistochemistry

*indicates *HER2* gene amplification by silver in situ hybridization

### Concordance of HER2 status between primary gastric adenocarcinomas and CB-MEs

**Fig. 1:**
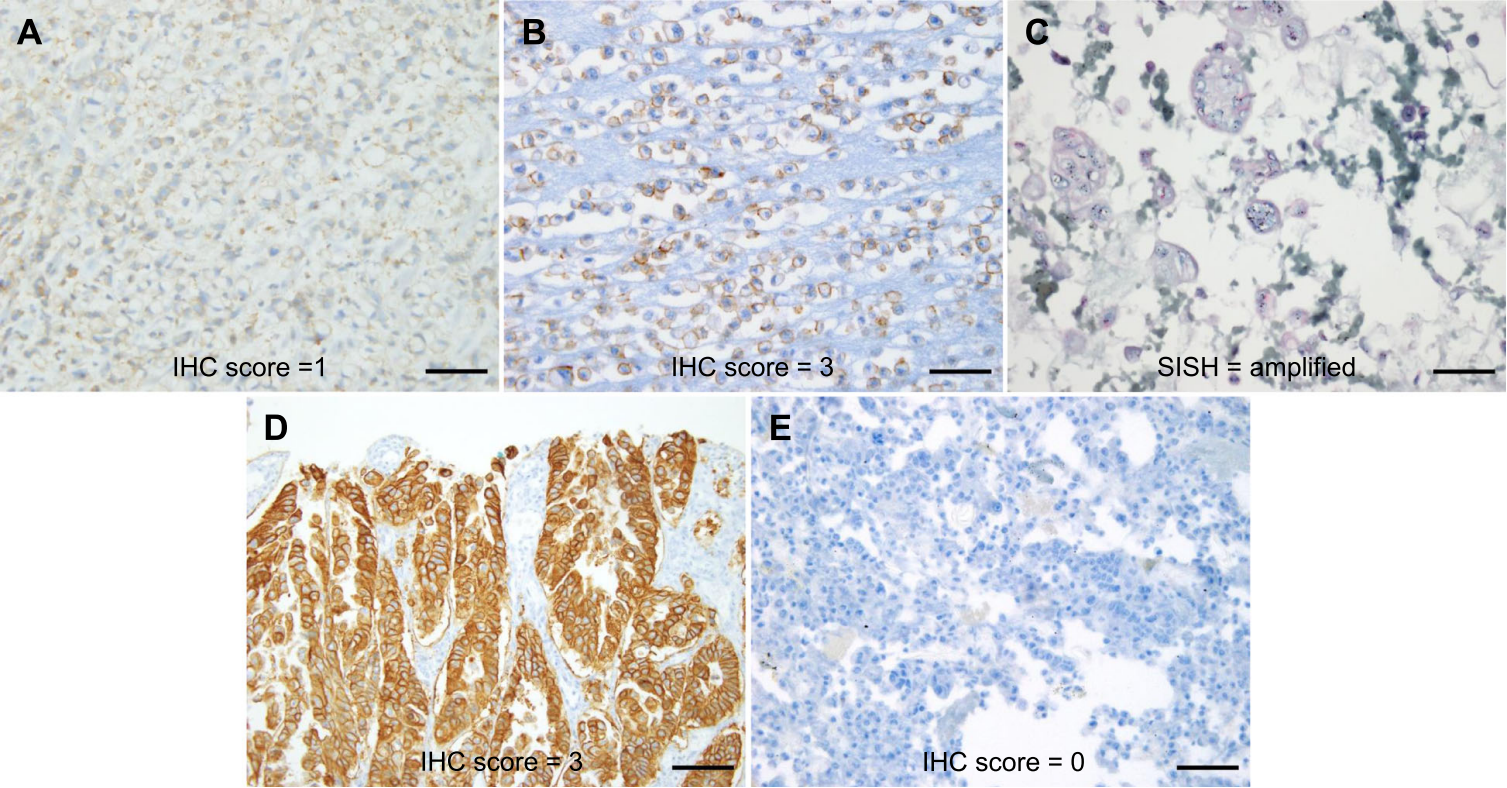
Representative cases with discordant HER2 status between the primary gastric adenocarcinoma tissue and the CB-ME. The endoscopic biopsy sample of the primary gastric adenocarcinoma from one patient showed HER2 negativity (**a**; IHC score, 1; original magnification, × 400; scale bar, 50 μm), but the CB-ME showed strong membranous staining (**b**; IHC score, 3; original magnification, × 400; scale bar, 50 μm), and *HER2* gene amplification was detected by SISH (**c**; original magnification, × 400; scale bar, 50 μm). In another patient, the endoscopic biopsy sample of the primary gastric adenocarcinoma showed strong HER2 expression (**d**; IHC score, 3; original magnification, × 200; scale bar, 100 μm), but the CB-ME showed no membranous reactivity in any tumor cell (**e**; IHC score, 0; original magnification, × 400; scale bar, 50 μm). HER2, human epidermal growth factor receptor 2; CB-ME, cell block of malignant effusion; IHC, immunohistochemistry; SISH, silver in situ hybridization

The comparison between HER2 positivity in primary gastric adenocarcinomas and CB-MEs is shown in Table 3. HER2 positivity was observed in 4.4% (2/45) of primary gastric adenocarcinomas and 6.7% (3/45) of CB-MEs. Overall, the HER2 status concordance rate between the primary gastric adenocarcinoma tissue and CB-MEs was 88.9% (40/45); however, the concordance level between the two sample types was evaluated as poor (κ = − 0.056), since the five HER2-positive primary gastric adenocarcinoma and CB-ME samples were from five different individuals for whom the paired sample was negative. Figure 1 shows representative cases of discordant HER2 status between the primary gastric adenocarcinoma and CB-ME from the same patient.

**Table 3:** Concordance of HER2 status between in the primary gastric adenocarcinomas and cell blocks of malignant effusions

HER2 status in primary tumor tissues	HER2 status in the cell blocks of malignant effusions		Total
	Negative	Positive	
Negative	40 (88.9%)	3 (6.7%)	43 (95.6%)
Positive	2 (4.4%)	0 (0.0%)	2 (4.4%)
Total	42 (93.3%)	3 (6.7%)	45 (100.0%)

*HER2*, Human epidermal growth factor receptor 2

### HER2 status in serially sampled CB-MEs

**Fig. 2:**
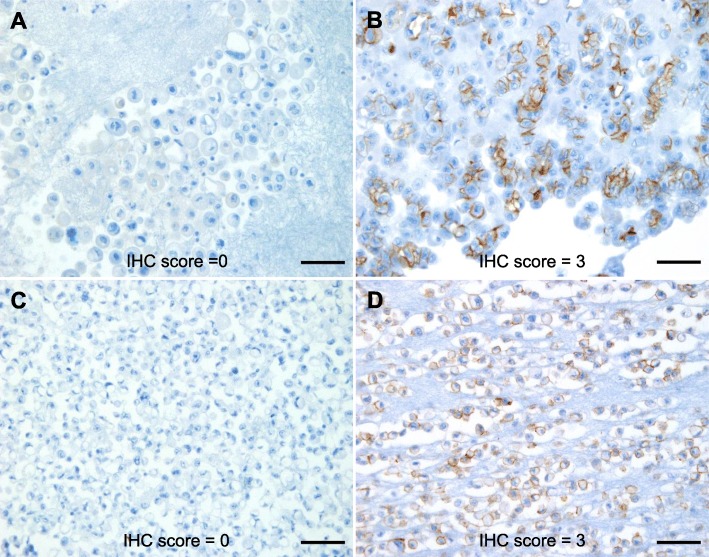
Representative cases of discordant HER2 IHC scores between serially sampled CB-MEs from a single patient. CB-MEs showed no membranous reactivity in any tumor cell, IHC score 0 (**a**; original magnification, × 200; scale bar, 100 μm; and **c**; original magnification, × 400; scale bar, 50 μm), whereas other CB-MEs from the same patient showed strong membranous staining, IHC score 3 (**b**; original magnification, × 200; scale bar, 100 μm; and **d**; original magnification, × 400; scale bar, 50 μm). HER2, human epidermal growth factor receptor 2; IHC, immunohistochemistry; CB-ME, cell block of malignant effusion

Of the 45 patients, 15 had two or more serially sampled CB-ME specimens. Of these, the IHC scores differed between each sample in six patients (40%). The 3/45 patients with HER2 positivity in CB-MEs shown in Table 3 exhibited HER2 positivity in one of the serially sampled CB-MEs. Figure 2 shows representative cases of discordant HER2 status between serially sampled CB-MEs.

## Discussion

Previous studies dealing with HER2 heterogeneity in gastric cancer have mainly focused on the concordance of HER2 status between endoscopic biopsy and surgically resected specimens, or between primary tissues and metastatic tissues [7–9, 16–19]. In this study carried out on 45 paired samples from gastric adenocarcinoma patients, we evaluated concordance in HER2 status between the primary tumor tissue and corresponding malignant effusions such as ascites or pleural effusion. We found that not only was HER2 status discordant between the primary gastric adenocarcinoma and the corresponding CB-ME but also, in the majority, between serially sampled CB-MEs.

In the present study, the percentage of cases with HER2 positivity was less than reported in other studies [4–8], and HER2 positivity was observed in 4.4% of primary gastric adenocarcinoma tumor tissue samples, 6.7% of CB-MEs, and 11% of both. This is likely due to the relatively high proportion (51.1%) of the Lauren diffuse histological type in our study population. This type more commonly metastasizes to the peritoneum but shows less HER2 overexpression and/or amplification than the intestinal type [5, 6, 8]. Of the five patients with HER2 positivity in primary tumor tissue or CB-ME, four had intestinal type, which was well or moderately differentiated, and only one patient had diffuse type with poor differentiation. In addition, the relatively high proportion of the diffuse type in our series may have contributed to the HER2 discordance observed between the primary gastric adenocarcinoma tissue and CB-MEs, and among two or more serially sampled CB-ME specimens. According to previous studies of intra-tumoral HER2 heterogeneity in gastric cancer, HER2 heterogeneity is associated with diffuse or mixed Lauren histological subtypes [20, 21].

HER2 evaluation by IHC or ISH before trastuzumab administration is essential for clinical practice, and many practical guidelines recommend that patients with unresectable locally advanced, recurrent, or metastatic adenocarcinoma of the stomach or GEJ be tested for HER2 status in tumor tissues. However, as intra- or inter-tumoral heterogeneity of HER2 expression in gastric cancer is well known to cause false-negative interpretation [7–9, 16–19], more assiduous tissue analysis is required to increase the possibility of detecting HER2 positivity. HER2 testing should be repeated on the surgically resected tumor from patients with a HER2-negative biopsy specimen [7–9, 18]. Moreover, repeat HER2 assessment of the metastatic sites is recommended in patients whose primary tumor is initially HER2 negative [7–9, 16, 19]. However, many gastric cancer patients have inoperable lesions and the endoscopic biopsy becomes the only available specimen in which to assess HER2. Furthermore, re-biopsy at a metastatic site may not be easy and also could have unpleasant complications due to the invasiveness of the procedure. Sampling malignant effusion such as ascites or pleural effusion is a relatively simple, minimally invasive, and repeatable procedure, and testing of effusion samples offers the distinct advantage of basing the HER2 assessment on the metastatic and, presumably, most aggressive clone of the tumor. Many gastric cancer patients present at an advanced stage of disease, with up to 40% having metastatic spread to the peritoneum with malignant ascites [10]. The cell block method enables cytologic materials from malignant effusions to be used like tissue samples for IHC or molecular analysis. Recent studies of metastatic breast cancer showed that hormone receptor or HER2 assessment performed on formalin-fixed and paraffin-embedded cell blocks prepared from fine needle aspiration and serous fluid is reliable in predicting the expression of these markers [22, 23]. Wong et al. also assessed the feasibility of HER2 assessment in 46 malignant effusions from metastatic gastric carcinoma and found 100% concordance in HER2 status between paired CB-MEs and histological specimens [24]. However, although this study supported the feasibility of HER2 testing on CB-MEs, only 18 (39%) of the total 46 CB-MEs were compared with histological specimens to assess HER2 status concordance. Here, we evaluated the HER2 status in 45 paired primary gastric adenocarcinoma tumor tissues and pathologically confirmed CB-MEs. Although a high concordance rate between primary gastric adenocarcinomas and CB-MEs (88.9%) was observed, the concordance level was poor; all five patients with HER2 positivity in the primary tumor or a CB-ME had a negative result in the corresponding paired sample. Possible explanations for detecting the opposite result in each sample type of the pair is genetic drift or clonal selection of HER2 during the metastatic process, or it is a consequence of intra-tumoral heterogeneity of HER2. Perhaps more likely is that adenocarcinoma cells in primary gastric tissues or CB-MEs are limited. In three patients with positive conversion in the CB-ME, all primary gastric adenocarcinoma tissues were endoscopic biopsy specimens and all CB-MEs showed high tumor cellularity, whereas in two patients with negative conversion in the CB-ME, both CB-MEs showed low cellularity. These findings indicate that reassessment of HER2 status is particularly warranted in cases of CB-ME with high cellularity when the representative sample of the primary tumor initially tested is a HER2-negative endoscopic biopsy.

There are no guidelines as to how HER2 IHC should be scored in cytology material. The current published criteria, which were used in the present study, have been largely formulated and validated for histological samples [7, 8, 13, 14]. We scored HER2 IHC in CB-ME specimens according to the modified Hofmann scoring criteria for biopsy specimens [13]. However, this system, which requires the identification of a cluster of five cohesive tumor cells, is difficult to apply when the predominant malignant population is singly dispersed. A revision of the criteria for cytology specimens, allowing for dispersed tumor cells, should be considered, including possibly reducing the threshold for the required number of cells deemed to be a positive result. While it might seem a logical extension of the Hofmann scoring criteria to require at least five dispersed cells, it could be argued that fewer than five malignant cells with a convincing IHC 3+ reaction in a limited sample represents genuine HER2 overexpression [24]. Similarly, the overall *HER2* gene copy number is an important consideration when analyzing ISH samples, in addition to the *HER2*:CEP17 ratio. If the ratio suggests borderline amplification, the total copy number should also be considered because a high count (i.e., > 6 copies) may indicate *HER2* amplification. As a consequence, it is recommended that if there are > 6 copies of the *HER2* gene seen with a single probe then the sample is considered to have amplified *HER2* [8]. The method used for analyzing ISH samples for *HER2* amplification is also important. In the present study, we used bright-field SISH rather than fluorescence ISH (FISH), which is commonly used to evaluate *HER2* amplification. Compared with FISH, SISH is more “pathologist friendly” and allows the evaluation of *HER2* gene amplification in its morphological context. Although studies comparing FISH and SISH show concordance rates of 91–100%, and that both FISH and SISH are reliable for *HER2* amplification testing [25], it is widely accepted that SISH is likely to become the most appropriate methodology for gastric cancer [8, 9]. The use of bright-field over fluorescent methodologies is advantageous when examining heterogeneous staining because they enable parallel evaluation of the microscopic morphology, thus allowing HER2-positive tumor foci within a heterogeneous sample to be rapidly identified. Therefore, although there may only be a small number of tumor cells in the CB-ME, it is important to consider the possibility of HER2 positivity if strong membranous staining by IHC is seen, even in the CB-ME. Furthermore, SISH should be considered as preferable when performing ISH to confirm *HER2* gene amplification, especially in CB-ME samples.

The present study has some limitations. First, we could not analyze the correlation between the HER2 status discordance and response to trastuzumab, the rationale for the HER2 test. The overall incidence of HER2 positivity was too low in our study population, and trastuzumab was used in only two patients with HER2 overexpression in the primary gastric adenocarcinoma. One patient had a partial response and the other had progressive disease after first-line palliative trastuzumab-containing chemotherapy. However, a recent study reported that patients with gastric cancer that was initially HER2 negative but became HER2 positive in a repeat biopsy or metastatic site had similar treatment benefits from trastuzumab-containing chemotherapy as a historical control group with an initially HER2-positive primary tumor [16]. Therefore, trastuzumab-containing chemotherapy could be considered for patients with HER2 positivity only in the CB-ME. Second, 67.6% of the total CB-ME samples were obtained during or after chemotherapy, not at the time of diagnosis of metastatic gastric cancer before chemotherapy. HER2-positive tumor cells have been shown to have a higher chemosensitivity than HER2-negative cells [26, 27], implying that detection of HER2 in CB-MEs obtained after previous cytotoxic chemotherapy would be unlikely. However, HER2 status could also change with disease evolution or the therapeutic process [16, 27], and for these reasons we suggest that CB-MEs can be useful if re-evaluation of HER2 status during the course of the disease is required. Third, we evaluated only the discordance of one marker using HER2 expression; however, most patients in our study had HER2-negative primary gastric tumors and CB-MEs. There can be discordance in many molecular biomarkers other than HER2 between primary gastric tumors and CB-MEs. Therefore, comprehensive molecular analyses comparing primary gastric adenocarcinomas and corresponding CB-MEs will disclose the complex heterogeneity of HER2-negative tumors. Finally, this study is a retrospective analysis from a single institution with a limited number of participating patients. Additional analysis of a larger number of patients from other institutions is required to confirm our findings. Nonetheless, to our knowledge, this is the largest study to date to specifically address the issue of concordance between primary gastric adenocarcinoma tissue and the corresponding CB-ME, a specimen commonly obtained from patients with metastatic gastric cancer.

## Conclusions

In conclusion, we recommend in gastric cancer patients with malignant ascites or a pleural effusion that a repeat HER2 assessment of the CB-ME is considered when the initial primary gastric adenocarcinoma is HER2 negative. The possibility of detecting HER2 positivity can be improved if the primary gastric adenocarcinoma tissue is analyzed along with all available CB-MEs.

## Data Availability

The datasets used and/or analysed during the current study are available from the corresponding author on reasonable request.

## Abbreviations

CB-ME: Cell block prepared from malignant effusion
CEP17: Chromosome 17
FISH: Fluorescence in situ hybridization
GEJ: Gastroesophageal junction
HER2: Human epidermal growth factor receptor 2
IHC: Immunohistochemistry
SISH: Silver in situ hybridization
WHO: World Health Organization
